# Disentangling the dynamics of energy allocation to develop a proxy for robustness of fattening pigs

**DOI:** 10.1186/s12711-023-00851-w

**Published:** 2023-11-07

**Authors:** Guillaume Lenoir, Loïc Flatres-Grall, Rafael Muñoz-Tamayo, Ingrid David, Nicolas C. Friggens

**Affiliations:** 1 Université Paris-Saclay, INRAE, AgroParisTech, UMR Modélisation Systémique Appliquée aux Ruminants, 91120 Palaiseau, France; 2 GenPhySE, Université de Toulouse, INRAE, ENVT, 31320 Castanet Tolosan, France; 3 AXIOM, 37310 Azay-Sur-Indre, France

## Abstract

**Background:**

There is a growing need to improve robustness of fattening pigs, but this trait is difficult to phenotype. Our first objective was to develop a proxy for robustness of fattening pigs by modelling the longitudinal energy allocation coefficient to growth, with the resulting environmental variance of this allocation coefficient considered as a proxy for robustness. The second objective was to estimate its genetic parameters and correlations with traits under selection and with phenotypes that are routinely collected. In total, 5848 pigs from a Pietrain NN paternal line were tested at the AXIOM boar testing station (Azay-sur-Indre, France) from 2015 to 2022. This farm is equipped with an automatic feeding system that records individual weight and feed intake at each visit. We used a dynamic linear regression model to characterize the evolution of the allocation coefficient between the available cumulative net energy, which was estimated from feed intake, and cumulative weight gain during the fattening period. Longitudinal energy allocation coefficients were analysed using a two-step approach to estimate both the genetic variance of the coefficients and the genetic variance in their residual variance, which will be referred to as the log-transformed squared residual (LSR).

**Results:**

The LSR trait, which could be interpreted as an indicator of the response of the animal to perturbations/stress, showed a low heritability (0.05 ± 0.01), a high favourable genetic correlation with average daily growth (− 0.71 ± 0.06), and unfavourable genetic correlations with feed conversion ratio (− 0.76 ± 0.06) and residual feed intake (− 0.83 ± 0.06). Segmentation of the population in four classes using estimated breeding values for LSR showed that animals with the lowest estimated breeding values were those with the worst values for phenotypic proxies of robustness, which were assessed using records routinely collected on farm.

**Conclusions:**

Results of this study show that selection for robustness, based on estimated breeding values for environmental variance of the allocation coefficients to growth, can be considered in breeding programs for fattening pigs.

## Background

The pig industry faces new challenges that are related to rapidly changing environmental conditions, especially those due to global warming [1], and to growing societal concerns. For several decades, breeding objectives were mainly focused on increasing animal productivity (growth, feed efficiency, etc.), at the expense of non-productive functions, i.e., fitness [2, 3]. This unfavourable consequence could be explained by trade-offs in resource allocation between biological functions [4]. Indeed, when animals cannot obtain more resources, i.e., under limiting environments, allocation of these resources to a high priority function is detrimental to another function [5]. In this situation, the animal is unable to maximize the expression of each biological function simultaneously. This requires animals that are able to adapt to new environmental conditions with more limiting resources, which can be associated with an improvement in robustness that Knap [6] defined as “*the ability to combine a high production potential with resilience to stressors, allowing for unproblematic expression of a high production potential in a wide variety of environmental conditions”.* Generally, production potential is associated with a phenotype of interest, such as growth, feed efficiency, milk production, or egg production. This definition of robustness also integrates the concept of resilience, which can be defined as the ability of an animal to be minimally affected by a perturbation or to return to its initial state before the perturbation [7, 8]. Thus, incorporating one or several traits that evaluate the robustness of growing pigs in breeding programs would be of value for the development of more sustainable breeding goals [7]. However, when robustness is a breeding objective, it is important to simultaneously maintain a high level of production to meet the industry’s economic expectations.

Until recently, traits in breeding goals that may be associated with robustness are mainly related to resistance to diseases, mortality during a specific period, longevity of reproductive animals, or performance under suboptimal conditions [6–9]. Traits based on environmental sensitivity have also been proposed [10] (reaction norm or structural models of variance), but their development has been limited due to issues with the collection and processing of the data necessary for their implementation. Fortunately, in recent years, the increasingly common use of sensors on pig farms, especially automatic feeding systems (AFS), allows continuous individual recording of weight or feed intake over a long period and thus, analysis of the dynamics of those longitudinal measurements, offers the possibility to characterize the response of an animal facing a perturbation. Several studies have used such longitudinal data to quantify robustness or resilience indicators based on deviations of the observed trajectory of feed intake [11] or body weight [12] from their expectations in a non-perturbed environment. The challenge in these approaches, is the definition and modelling of expected trajectories of individual animals. Other studies have developed resilience indicators based on the within-individual variance of time series measurements related to production, including feed intake for growing pigs [13], milk yield for dairy cows [14] and egg production for laying hens [15]. These approaches have mainly characterized robustness or resilience through analysis of one production variable. They represent a substantial contribution to the phenotyping of resilience but do not address the underlying biological mechanisms and the potential trade-offs in the use of available resources between production and other functions. A robust animal can be considered as an animal that is able to allocate a proportion of its resources to the right function at the right time [16]. To our knowledge, characterization of robustness based on the temporal evolution of the allocation pattern has been little explored in pigs.

Acquisition of temporal data on feed intake and weight in growing pigs has made it possible to consider the development of an allocation model based on these two variables to characterize robustness. With this objective, we recently developed a conceptual model to represent the temporal pattern of the allocation of energy intake to growth in fattening pigs [17]. Using this model, our first objective in the present study was to develop and evaluate a robustness indicator based on the modelling of longitudinal energy allocation coefficients to growth in fattening pigs. The residual variance of these allocation coefficients was considered as a proxy for robustness, as it is expected to reflect the ability of an animal to express or adapt its production potential in the face of changes in its environment compared to that of other animals that have been raised under the same conditions. Our objective was to estimate the heritability of this proxy and its genetic correlations with traits under selection and with other phenotypes that are routinely collected and that are associated with robustness or health status.

## Methods

### Study population

In total, 25,745 pigs from the Pietrain NN Français paternal line (Pie NN) of the AXIOM company, i.e. that do not carry the halothane sensitivity allele, were used in this study. Individuals from the Pie NN line were born on three farms that are integrated into the AXIOM breeding scheme and that comply with AXIOM’s biosafety and health requirements. Some of the males were selected before weaning and raised on the boar test station of the breeding company AXIOM Genetics (Azay-sur-Indre, France). The animals considered in the present dataset included 6885 entire males and 13,012 females that were raised and individually tested on their farm of birth from April 2014 to April 2022, and 5848 entire males that were raised from September 2015 to April 2022 on the boar test station.

The animals raised on their farm of birth were born from 3943 litters, with 6.5 ± 2.9 (standard deviation) piglets per litter, and from 321 sires, with 80 ± 53.8 piglets per sire. To limit the risk of confounding between environmental (i.e. fattening group) and genetic effects, the sires were used at least in two mating groups on each farm and on two farms. The animals were transferred to fattening rooms when they were 75.7 ± 3.4 days old [33.8 ± 7.8 kg body weight (BW)] and kept for 68.6 ± 4.9 days until individual testing at around 142.4 ± 4.6 days of age (103.4 ± 11 kg BW). On the three farms, each fattening group included 65.7 ± 22.8 females and 42.1 ± 18.2 males.

Males that were raised on the boar test station were transferred every 3 weeks from the farm of birth to the station, at an average age of 27.3 ± 2.2 days and an average BW of 8.5 ± 1.7 kg. They were raised in pens of 14 animals from the same farm of birth, which remained unchanged at each rearing stage. Each fattening group consisted of 62.4 ± 20.7 animals that came from one to three farrowing farms and from 2048 litters, with 2.6 ± 1.5 piglets per litter, and were born from 238 sires, with 22.1 ± 15 piglets per sire. Pigs were raised in quarantine and post-weaning rooms for 5 and 2 weeks, respectively, and transferred to fattening rooms when they were 76.4 ± 2.9 days old (34.4 ± 5.4 kg BW) for 69 ± 4.7 days until individual testing at the age of ~ 145.4 ± 3.6 days (104.5 ± 11.1 kg BW). Fattening rooms of the testing station were equipped with Nedap pig performance testing feeding stations (Nedap N.V.; Groenlo, the Netherlands). Animals were fed ad-libitum with commercial diets that were adapted to their physiological needs and formulated to be non-limiting in amino acids. The environmental and technical conditions on the boar test station were described in detail in Lenoir et al. [18].

#### Information recorded during the fattening period

The same phenotypes were recorded on the farrowing farms and the boar test station. Each animal was individually weighed upon arrival in the fattening room (initial body weight: IBW). When the average weight of the group was approximately 100 kg, at an average age of 144 ± 4.9 days, performance tests were conducted on animals that weighed more than 70 kg [19]. Animals that weighed less than 70 kg were excluded from the individual test because of poor growth rate, as defined by the French Pork and Pig Institute in the specifications for on-farm testing [19]. The following measurements were recorded on animals that weighed more than 70 kg: body weight (TBW), average ultrasonic backfat thickness (BF, average of three measurements in mm), and ultrasonic *longissimus dorsi* thickness (LD, one measurement in mm). The BF and LD measures were adjusted to 100 kg liveweight (BF100 and LD100, respectively) in order to compare the animals at an equivalent weight. For these adjustments, it was assumed [20] that the rates of change in BF and LD per kg liveweight were: 0.04 mm/kg and 0.27 mm/kg, respectively. Average daily gain (ADG) was calculated as the difference between TBW and IBW divided by the number of days elapsed between the two weigh dates.

In addition, on the boar testing station, BW (kg) and feed intake (FI; kg per visit) were recorded each time the animal went into the AFS, including pigs that weighed less than 70 kg on the day of individual testing. Feed conversion ratio (FCR) was calculated as the ratio between total FI during the fattening period and weight gain (TBW-IBW), expressed in kg/kg. Average daily feed intake (DFI) was calculated as the total FI during the period divided by the number of days elapsed. Residual feed intake (RFI) was estimated for each animal as the deviation between the recorded DFI and the predicted average daily feed intake (PDFI) based on requirements for maintenance and production. Based on the method proposed by Labroue et al. [21], PDFI was estimated by linear regression of DFI on average metabolic weight (AMW), ADG, and BF100, using the *lm* function in R [22]. The AMW was estimated for each animal using the formula proposed by Noblet et al. [23] as ${\text{AMW}} = \frac{{\left( {{\text{TBW}}^{1.6} - {\text{IBW}}^{1.6} } \right)}}{{1.6\left( {{\text{TBW}} - {\text{IBW}}} \right)}}$. All individual medical treatments received by each animal were also recorded. At the time of testing, visual observation of each animal was carried out by the technician in charge of measurements in order to note any morphological defects, anomalies, and clinical signs of disease according to a frame of reference [19], noted as “observable defects”. The technician was the same person for a given fattening group. Medical treatments and individual observations were recorded from January 2019 to April 2022 on 3028 males on the boar test station. The pedigree contained 27,276 animals over 20 generations.

#### Longitudinal data quality control and processing

A quality control process was applied to the BW and FI data that were recorded each time the animal went into the AFS, to validate the data, identify quality issues, and accumulate them on a daily scale, as proposed by [12] and modified by [18]. This procedure and different exclusion thresholds were defined to exclude measurement errors, related to technical issues of the AFS, without excluding intra and inter-individual variability. In brief, using the BW visit data from a given animal, a quadratic regression of BW on age + age^2^ was applied to eliminate aberrant BW values. For a given animal and a given visit, if the absolute value of the ratio between the residual value and the fitted value was greater than 0.15, the BW measurement was considered as missing. After repeating this step for a second time, the body weight (${\text{BW}}_{it}$; kg) was estimated from the median of the non-missing weights for each pig ($i$) and for each day since the transfer to the fattening room ($t$). For feed intake, if for a given animal the feed intake rate at a visit was lower or higher than its mean intake rate over the fattening period ± 4 standard deviations, the FI measurement was set to be missing and imputed using a linear regression of FI on feeding duration. Daily feed intake of a given pig on a given day (${\text{FI}}_{it}$; kg) was calculated as the sum of intakes during the visits on that day. Then, ${\text{BW}}_{it}$ and ${\text{FI}}_{it}$ were validated at the pen scale to detect inconsistencies associated with the AFS equipment in the pen. When a control day was missing (due to a mechanical problem of the AFS or loss of a RFID tag), the missing ${\text{BW}}_{it}$ (2.8% of the records) and ${\text{FI}}_{it}$ (0.8% of the records) were each imputed using the local regression model “*proc loess*” implemented in SAS [24]. Data recorded on day of entry into the fattening room were excluded from the dataset due to calibration of the AFS and animal adaptation. Animals that were evaluated for at least 20 consecutive days were kept, even if they died during the test period or weighed less than 70 kg on the day of testing. After processing of the data, the file included 405,983 daily records for the 5848 males that were fattened on the test station, representing 91.9% of all animals in the original data.

### Models for analysis

#### Modelling the energy allocation coefficient to growth

**Fig. 1:**
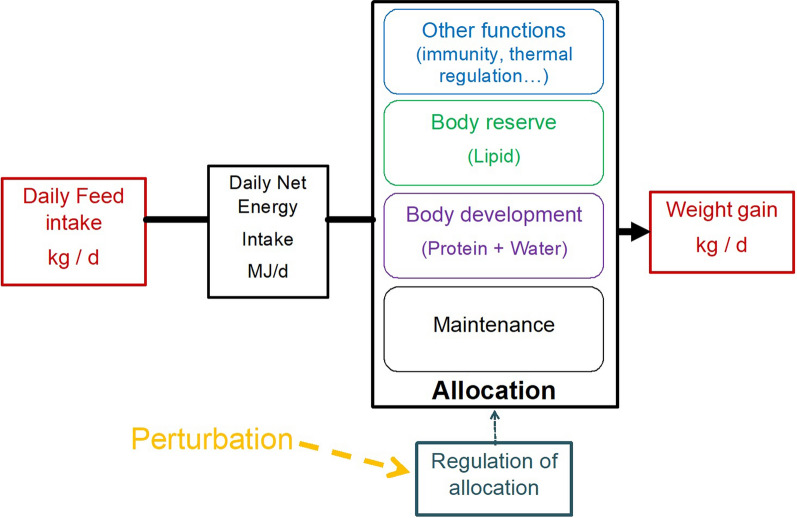
Conceptual model of resource allocation in growing pigs. In red: variables recorded by AFS

To estimate the energy allocation coefficient to growth, we propose a conceptual approach to model the relationship between feed intake and growth. We considered that feed intake, i.e., the input of the system, is transformed into net energy and allocated to several functions: maintenance, body development (protein deposition), body reserves (lipid deposition), and other functions [25, 26]. Body weight gain, i.e., the output of the system, is directly related to protein and lipid deposition [27]. Resource allocation to these functions is assumed to be regulated during the fattening period according to the individual’s genetic potential and degree of maturity [28]. Over time, the resource allocation coefficient is also impacted by changes in environmental conditions, i.e., perturbations [16]. Figure 1 provides a simplified layout of this model.

A dynamic linear regression model (DLM) [29] was used to estimate the daily energy allocation coefficient to growth ($\upalpha _{it}$) [17]. First, ${\text{FI}}_{it}$ was converted into net energy intake in MJ (${\text{EI}}_{it}$), using the net energy density of the feed, i.e. 9.85 MJ of NE/kg. Then, the net energy available for growth at day $t$ (${\text{NEA}}_{it}$) was calculated as the difference between ${\text{EI}}_{it}$ and the net energy maintenance requirements at day $t$ (${\text{MR}}_{it}$), which were estimated according to Noblet et al. [27]. The DLM to estimate the allocation coefficient of energy to weight gain for a given pig $i$ at day $t$ (${\upalpha }_{it}$) was built using two equations: an observation Eq. (1) that relates cumulative weight gain on day $t$ since $t = 0$ (${\text{CW}}_{it}$ in kg) with cumulative net energy available on day $t - 1$ (${\text{CNEA}}_{it - 1}$ in MJ); and a system Eq. (2) that describes changes in ${\upalpha }_{it}$ (an unobserved state variable) from day to day according to a stochastic process:

1 $${\text{CW}}_{it} = \upalpha _{it} *{\text{CNEA}}_{it - 1} + v_{it} ,\; {\text{with}}\;{\mathbf{v}}_{{\mathbf{i}}} \sim {\text{N}}\left( {0,{\text{I}}\upsigma _{iv}^{2} } \right),$$

2 $$\upalpha _{it} = \upalpha _{it - 1} + w_{it} ,\; {\text{with}}\;{\mathbf{w}}_{{\mathbf{i}}} \sim {\text{N}}\left( {0,{\text{I}}\upsigma _{iw}^{2} } \right),$$

where $v_{it}$ is a random observation error for animal $i$; ${\upsigma }_{iv}^{2}$ is the observational variance for animal $i$; $w_{it}$ are the random and unpredictable changes in level between time $t - 1$ and $t$; and ${\upsigma }_{iw}^{2}$ is the system variance. Compared to daily weight gain and intake, the use of cumulative weight gain and intake has the advantage of being less impacted by the presence of noise in measurements and by the effects linked to the dates of measurements, and of allowing easier representation of individual trajectories [11, 30]. This model was built using the R *package dlm* [31]. Estimation of the unobserved state variable at time $t$ was carried out using the Kalman smoother, a recursive algorithm that uses all available information [29]. The values of ${\upalpha }_{it}$ were estimated independently for each animal. The value of ${\upalpha }_{it}$ at $t = 1$ was not estimated because the consumption at $t - 1 = 0$ was unknown.

#### Estimation of the genetic variance in environmental variance

Individual estimates of longitudinal energy allocation coefficients (${\upalpha }_{it}$) were analysed with the ASReml 4.2 software [32] using a two-step approach [33, 34] to estimate both the genetic variance of the allocation coefficients and the genetic variance of the residual variance (i.e., environmental variance).

##### Step (1): estimation of the genetic variance of the energy allocation coefficients

The individual estimates of the energy allocation coefficient were analysed using a random regression model (RR) with first order Legendre polynomials [35] for the genetic and permanent environmental effects:

3 $$\begin{aligned} \alpha_{ijklmt} & = \mu + batch_{j} + pen_{l} .batch_{j} + age_{it} \\ & \quad + \mathop \sum \limits_{k = 0}^{1} a_{ki} \varphi_{k} \left( t \right) + \mathop \sum \limits_{k = 0}^{1} p_{ki} \varphi_{k} \left( t \right) + litter_{m} + \varepsilon_{ijlmt} , \\ \end{aligned}$$

where $\alpha_{ijklmt}$ is the energy allocation coefficient of pig $i$ at age $k$ at time $t$, that was born in litter $m$ and raised in fattening group $j$ and in pen $l$, and $\mu$ is the population mean. The fixed effects included in the model were selected at an α-risk of 5% using the Wald F statistic and included the fattening group $batch_{j}$ (103 levels) as contemporary group, the joint effect of fattening group and pen, $pen_{l} .batch_{j}$ (517 levels), and $age_{it}$ in days of the animal at day $t$ as a covariate. The common litter effect, $litter_{m} ,$ was included as a random effect (2048 levels). Significance of random effects was tested using a likelihood ratio (LRT) test, with an $\alpha$*-risk* of 5%. Furthermore, $a_{ki}$ is the random additive genetic effect and $p_{ki}$ the random permanent effect of pig $i$ at age $k$. Functions $\varphi_{k}$ are the Legendre orthogonal polynomials of degree $k$ [35]. The distributions of random effects were assumed to be normal:

$$\left[ {\begin{array}{*{20}c} {{\mathbf{a}}_{{\mathbf{0}}} } \\ {{\mathbf{a}}_{{\mathbf{1}}} } \\ \end{array} } \right] \sim \user2{ }N\left( {{\mathbf{0}},\user2{ }{\mathbf{G}} \otimes {\mathbf{A}}} \right),$$

$$\left[ {\begin{array}{*{20}c} {{\mathbf{p}}_{{\mathbf{0}}} } \\ {{\mathbf{p}}_{{\mathbf{1}}} } \\ \end{array} } \right] \sim \user2{ }N\left( {{\mathbf{0}},\user2{ }{\mathbf{P}} \otimes {\mathbf{I}}} \right),$$

where ${\mathbf{G}}$ and ${\mathbf{P}}$ are the matrices of, respectively, the additive genetic and permanent environmental variances and covariances of random regression coefficients; ${\mathbf{A}}$ is the pedigree-based relationship matrix, based on a pedigree of 27,276 animals over 20 generations, and ${\mathbf{I}}$ is an identity matrix of the order corresponding to the permanent environmental effects. The residual variance was assumed constant over time. Hence, the analysis was performed assuming a homoscedastic model.

##### Step (2): estimation of the genetic variance of the residual variance

In the second step of the analysis, the log-transformed square of the estimate of the residual from Model 3 (i.e. from $\varepsilon_{it}$), i.e. $ln\left( {\varepsilon_{it}^{2} } \right)$, were calculated for each individual $i$ and at each timepoint $t$, and will be referred to as LSR hereafter. A lower LSR value at day $t$ is assumed to indicate a greater robustness of an animal to environmental perturbations, which is related to a smaller deviation from the expected allocation of energy to growth. To follow the assumption of the best linear unbiased prediction (BLUP) [36] method of a non-selected base population, and to estimate the covariance between traits, a multi-trait animal model for the four traits under selection (ADG, BF100, LD100 and FCR, with a single measurement for each of the animals) and the non-selected traits LSR (repeated data for animal in station) and RFI was used for genetic parameter estimation. For LSR, which is calculated for each day $t$, the same fixed effects were fitted as for ${\upalpha }_{it}$ (see Eq. 3) and the random effects included litter, permanent environment, and animal additive genetic effects. For the four traits under selection, the fixed effects that were significant at an α-risk of 5% using the Wald F statistic were gender (2 levels), fattening farm (4 levels), and fattening group within the fattening farm (443 levels). Significant random effects were litter and animal additive genetic effects. Based on estimates from this step, heritability (h^2^) was estimated as the ratio of the estimates of animal genetic variance to the phenotypic variance, i.e., the sum of estimates of the genetic additive variance, common litter variance, permanent environment variance, and residual variance.

#### Relation between LSR and routinely collected phenotypes

To evaluate whether the LSR phenotype can be considered as a proxy for robustness, the relationships between estimated breeding values (EBV) for LSR and health phenotypes were estimated. The 3028 males with LSR phenotype and available observational and medical treatment information during the fattening period were divided into four quartiles according to their EBV for LSR, from Q1 for the most favourable values (lower EBV for LSR) to Q4 for the most unfavourable values (higher EBV for LSR). We studied the distribution of the phenotypes that were derived from measurements recorded during the animal performance evaluations, and from the medical treatments that were recorded during the test period to create two scores. This was done for the four classes of EBV for LSR (one for each quartile of animals). In each LSR EBV class, we differentiated animals that could be selected (Selectable or not) from those that were dead, or that weighed less than 70 kg on the day of individual testing, or that weighed 70 kg or more and with an observable defect on the day of testing. We considered factors such as poor development or other observable defects on the day of testing (Appendix) that were considered to be related to the robustness of the animal. A second score differentiated the pigs that had received at least one individual antibiotic or anti-inflammatory injection during the test period from those that had not received any injection (No injection). We also differentiated pigs that were “Selectable” without receiving any antibiotic or anti-inflammatory injection during the test period (Selectable without injection) from the others. Two by two Chi-square tests were used to compare the differences in frequencies of the scores among the four classes of EBV for LSR. Statistical significance was set a priori at a p-value less than or equal to 0.05.

## Results

### Estimated allocation coefficients and associated robustness indicator

The descriptive statistics for the dataset used in this study are in Table 1. The averages of the estimates of residual variances of the DLM were 113.2 ± 77.5 kg^2^ for ${\upsigma }_{iv}^{2}$ in the observation equation and 0.00031 ± 0.00045 kg^2^/MJ NE^2^ for ${\upsigma }_{iw}^{2}$ in the system equation. The means of ${\upalpha }_{t}$ and LSR were 0.099 ± 0.027 kg/MJ NE and − 12.62 ± 2.50, respectively. The phenotypic correlations, estimated with the *cor.test* function in R [22], for trait ${\upalpha }_{t}$ were positive with $e_{t}$ (0.241 ± 0.002) and with LSR (0.23 ± 0.002), which means that a higher energy allocation rate to growth was associated with greater variability, i.e., lower robustness. The phenotypic coefficients of variation were greater than 20% for IBW, ${\upalpha }_{t} ,$ and LSR, and between 10 and 20% for TBW, ADG, DFI, and BF100, which indicate large phenotypic variations for all these traits.

**Table 1:** Descriptive statistics of the variables recorded or estimated on fattening pigs

Trait (unit)	Number of animals/records if repeated measures	Mean	SD	Coefficient of variation
IBW (kg)	25,745	33.8	7.8	23.1%
TBW (kg)	25,365	103.7	11.1	10.7%
ADG (kg/d)	25,322	0.977	0.109	11.1%
FCR (kg/kg)	8675	2.25	0.21	9.3%
DFI (kg/d)	8675	2.19	0.29	13.2%
RFI (kg/d)	8675	− 0.005	0.169	–
BF100 (mm)	25,323	7.66	1.19	15.5%
LD100 (mm)	25,320	68.26	6.34	9.3%
${\upalpha }_{t}$ (kg/MJ)	5848/405,104	0.099	0.027	27.3%
$e_{t}$ (kg/MJ)	5848/405,104	0	0.0096	–
LSR	5848/405,104	− 12.62	2.50	19.8%

*IBW* initial body weight, *TBW* terminal body weight, *ADG* average daily gain, *FCR* feed conversion, *DFI* daily feed intake, *RFI* residual feed intake, *BF100* backfat thickness estimated at 100 kg liveweight, *LD100* longissimus dorsi thickness estimated at 100 kg liveweight, ${\upalpha }_{t}$ allocation coefficient to growth, $e_{t}$ residual of RR model, *LSR* log-squared residual, robustness indicator

**Fig. 2:**
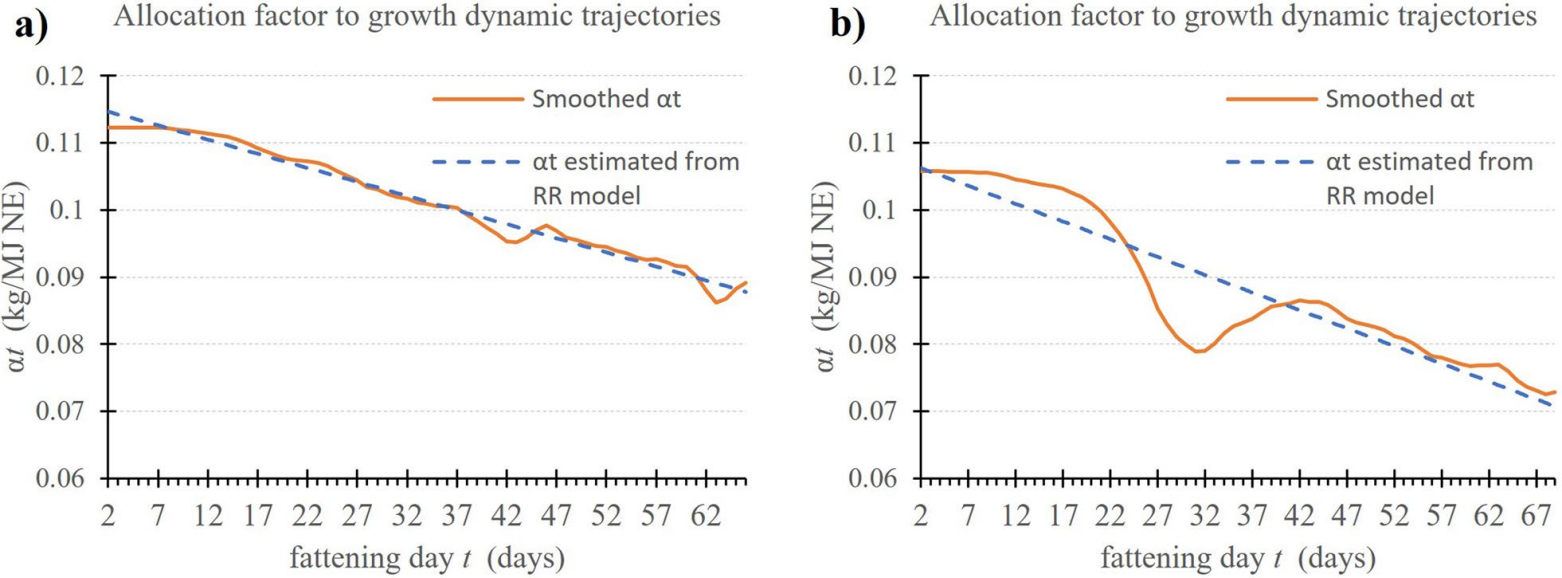
Example of two dynamic trajectories of the allocation coefficients α_*t*_ during the fattening period for two animals: smoothed with the dynamic linear model (DLM) (orange line) and its prediction from the random regression (RR) model (blue dotted line)

Figure 2 shows the ${\upalpha }_{t}$ trajectories of two animals that exhibited different patterns. The first animal in Fig. 2a had a smooth allocation trajectory over time, close to its prediction from the RR model, and its average LSR value was − 14.6 ± 1.7. The second animal in Fig. 2b had a greater deviation between the smoothed allocation and its prediction, which is likely a response to an environmental perturbation. The average LSR value of the second animal was higher than that of the first individual (− 12.3 ± 1.9). Accordingly, the parameter LSR appears to be a useful indicator to quantify the effect of a perturbation on an animal and allows comparisons within a population.

### Genetic parameters of the allocation coefficients, production, and robustness indicator traits

**Fig. 3:**
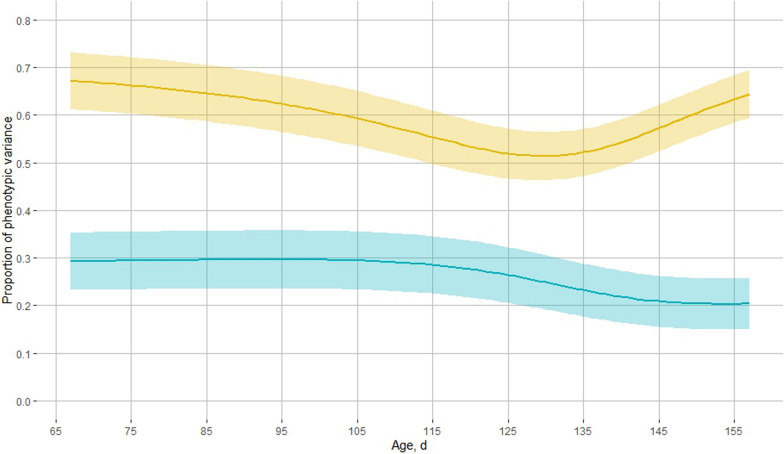
Estimates of heritability (${h_{k}}^{2}$; blue) and permanent environmental effects (${p_{k}}^{2}$; yellow) for the energy allocation coefficient α_*t*_ across age in days under the random regression model (RR) using Legendre orthogonal polynomials. Shaded area: 95% confidence interval

Estimates of heritability for ${\upalpha }_{t}$ over time based on the RR model are shown in Fig. 3 and ranged from 0.20 ± 0.03 to 0.30 ± 0.03. Estimates of heritability were stable from 67 to 100 days of age, i.e. around 0.30 ± 0.03, then decreased up to 150 days of age and stabilized to around 0.20 ± 0.03 towards the end of the test period. Estimates of permanent environmental variance proportions ranged from 0.51 ± 0.03 to 0.64 ± 0.03 and decreased up to 128 days of age and then increased again towards the end of the test period.

The heritability estimates for the traits under selection, ADG, BF100, LD100, and FCR, were moderate, ranging from 0.27 ± 0.03 to 0.45 ± 0.02 (Table 2), and those for RFI and FCR were not significantly different from each other, 0.29 ± 0.03 and 0.27 ± 0.03, respectively. The robustness indicator LSR was lowly heritable, i.e. 0.05 ± 0.01. The proportion of variance due to common litter effects was similar for all traits, ranging from 0.04 ± 0.01 to 0.06 ± 0.01, except for an estimate close to 0 for LSR. The proportion of phenotypic variance explained by the permanent environment effect for LSR was moderate at 0.22 ± 0.01.

**Table 2:** Estimates of heritability (h^2^), common litter effect ratio (c^2^), permanent environmental effect ratio (p^2^), and phenotypic (Vp) and additive genetic (Va) variances for the recorded traits (± standard error)

Trait	h^2^	c^2^	p^2^	Vp	Va
BF100	0.45 ± 0.02	0.04 ± 0.01	–	1.02 ± 0.02	0.46 ± 0.03
LD100	0.29 ± 0.02	0.04 ± 0.01	–	15.78 ± 0.20	4.54 ± 0.33
ADG	0.37 ± 0.02	0.06 ± 0.01	–	0.0100 ± 0.0001	0.0037 ± 0.0002
FCR	0.27 ± 0.03	0.04 ± 0.01	–	0.0222 ± 0.0004	0.0061 ± 0.0006
RFI	0.29 ± 0.03	0.04 ± 0.01	–	0.0343 ± 0.0066	0.0099 ± 0.0010
LSR	0.05 ± 0.01	0.004 ± 0.004	0.22 ± 0.01	5.55 ± 0.03	0.25 ± 0.04

*BF100* backfat thickness estimated at 100 kg liveweight, *LD100* longissimus dorsi thickness estimated at 100 kg liveweight, *ADG* average daily gain, *FCR* feed conversion ratio, *RFI* residual feed intake, *LSR* log-squared residual, robustness indicator

The LSR trait had high negative estimates of genetic correlations with ADG, FCR, and RFI, ranging from − 0.83 ± 0.06 to − 0.71 ± 0.06 (Table 3). The estimates of the genetic correlations of LSR were low and negative with BF100 and not significantly different from 0 with LD100. The trait FCR had a high genetic correlation estimate with RFI, 0.90 ± 0.02, and moderate genetic correlation estimates with ADG (0.52 ± 0.06) and BF100 (0.50 ± 0.05). The estimates of the genetic correlations of ADG with BF100 and RFI were positive and moderate to high, i.e. 0.43 ± 0.04 and 0.61 ± 0.05, respectively.

**Table 3:** Estimates of genetic correlations (r^2^a ± standard error) of the robustness trait (LSR) with production traits

Trait	LD100	ADG	FCR	RFI	LSR
BF100	− 0.13 ± 0.05	0.43 ± 0.04	0.50 ± 0.05	0.32 ± 0.06	− 0.19 ± 0.07
LD100		− 0.24 ± 0.05	− 0.09 ± 0.05	− 0.08 ± 0.07	0.02 ± 0.07
ADG			0.52 ± 0.06	0.61 ± 0.05	− 0.71 ± 0.06
FCR				0.90 ± 0.02	− 0.76 ± 0.06
RFI					− 0.83 ± 0.06

*BF100* backfat thickness estimated at 100 kg liveweight, *LD100* longissimus dorsi thickness estimated at 100 kg liveweight, *ADG* average daily gain, *FCR* feed conversion ratio, *LSR* log-squared residual, robustness indicator

### Relationships between LSR EBV classes and recorded phenotypes

**Fig. 4:**
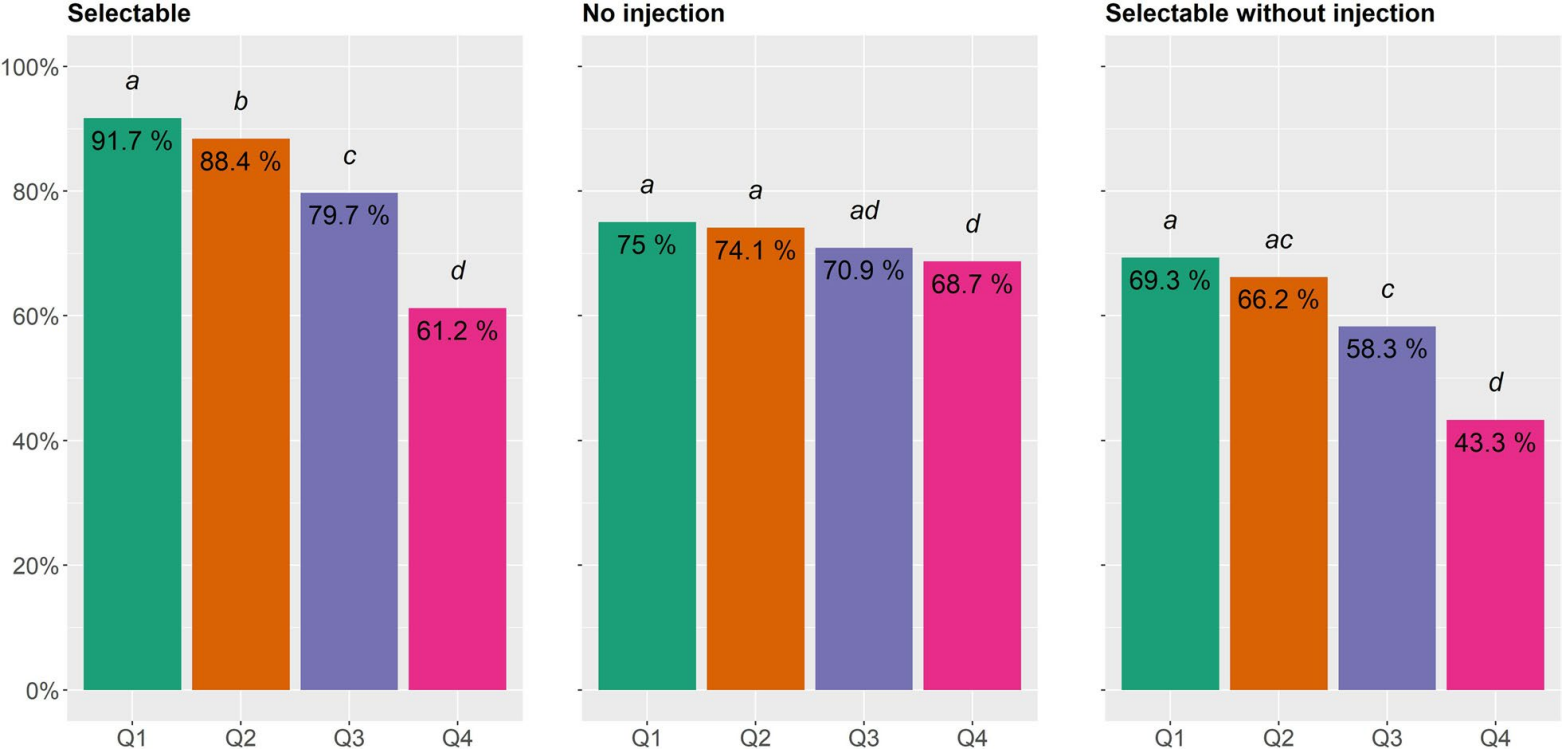
Distribution of percentages of pigs that can be selected (Selectable), that did not receive antibiotic or anti-inflammatory injections (No injection) or that were “Selectable” without receiving antibiotic or anti-inflammatory injections during the test period (Selectable without injection) depending on the quartile of their estimated breeding value (EBV) for the robustness indicator (LSR). Q1: pigs with the lowest LSR EBV, i.e., higher robustness genetic potential; Q4: pigs with the highest LSR EBV, i.e., lower robustness genetic potential. Bars with different letters are significantly different (P < 0.05)

The percentage of “Selectable” animals was significantly related with LSR EBV quartile (Fig. 4). The Q1 quartile, which included animals with the lowest EBV for LSR, i.e., the most robust animal, had the highest percentage of “Selectable” animals, i.e. 91.7%, and the Q4 quartile had the lowest, i.e. 61.2%. The differences in percentage of “Selectable” animals between each quartile were significant. In the Q1 quartile, 75% of the animals had not received any antibiotic or anti-inflammatory injection (“No injection”) over the control period. This percentage was not significantly different than those observed for the Q2 and Q3 quartiles, 74.1 and 70.9%, respectively. The difference in the percentage of animals with “No injection” between the Q4 and the Q1 or Q2 quartiles was significant at 68.7%. The proportion of animals “Selectable without injection” was significantly higher in the Q1 than the Q3 and Q4 quartiles, i.e. 69.3, 58.3, and 43.3%, respectively. In summary, a lower EBV for LSR, i.e., a higher level of robustness, was associated with a better chance of being in good health, being “selectable”, and having lower medical treatments.

## Discussion

Our objective was to propose a robustness indicator for fattening pigs based on the characterization of the energy allocation of the animal. This indicator is expected to be associated with the ability to cope with different types of environmental perturbations encountered, allowing optimal expression of the production potential. The originality of this work lies in the use of two time-series measured variables to model a longitudinal energy allocation coefficient, ${\upalpha }_{t}$, over the fattening period. The LSR trait was estimated as the daily difference in ${\upalpha }$ between the estimated values calculated with the DLM and the estimates from the RR model. Then, we studied the genetic background of LSR in order to assess its potential as a selection trait for robustness in fattening pigs. Our results indicate that LSR is a low heritability trait and has a strong favourable genetic correlation with growth and an unfavourable genetic correlation with FCR and RFI.

### Energy allocation to growth, from concept to model

When faced with one or more environmental disturbances, we can assume that a fattening pig has two types of responses: a change in feed intake pattern or a modification in energy allocation, i.e. a trade-off. These modifications in feed intake or in allocation patterns may or may not affect the pattern of body weight gain of the animal. This study focused on the second hypothesis, i.e. environmental disturbances affect the allocation pattern, with the objective to quantify robustness using a proxy estimated from variations in the energy allocation to growth over time. To our knowledge, this approach has not been studied in pigs from a selection purpose.

The effects of environmental conditions on feed intake have been widely studied in pigs, mainly the effects of temperature [37] and diseases [38]. Quantification of robustness or resilience through analysis of variations in feed intake has also been studied [11, 13, 39]. With respect to robustness, the effect of disturbances on growth patterns has been studied on pigs after weaning [40] or during the finishing period [12]. The present study complements the aforementioned studies by quantifying robustness through the prism of variation in a combination of two performance measures expressed as the allocation coefficient, which has been suggested to be an important biological component of robustness [4, 5].

Conceptually, for a fattening pig, it can be assumed that net energy is allocated to several functions: maintenance, growth (daily protein and lipid deposition), and other functions such as health or thermoregulation (Fig. 1). We can assume that the proportion of the available net energy that is allocated to each function is regulated by a “valve”, which increases or decreases allocation to each function over time. This hypothesizes that regulation in the allocation of the net energy is on the one hand driven by a “desired allocation” that depends on the characteristics of the individual (genotype, age), and on the other hand by “allocation permitted by the environment”.

The model structure employed here, as described in Fig. 1, does not include all details of the full process but provides a simple and biological way to represent energy allocation. Based on these assumptions and on data available in the context of the study, we built the model to estimate the allocation coefficient to growth at time $t$, ${\upalpha }_{t}$, based on daily feed intake and live weight measurements over time. Energy allocation to maintenance was estimated from metabolic body weight based on the equation proposed for an average animal by Noblet et al. [41], noting that this ignores differences between sexes, breeds, and individuals. Mobilization of lipid reserves, which allows for an increase in net energy available, was not integrated into the model. Indeed, mobilization of body reserves, apart from glycogen, is rare in growing animals [42]. Because it is not possible, in a large population, to evaluate precisely for a given pig at a given time, the net energy allocated to maintenance, to additional thermoregulation or physical activity, to protein deposition, and to lipid deposition, we used a pragmatic approach to estimate the energy available for growth at time $t$.

In this project, the animals were young and they were studied during a relatively short period compared to the lifespan scale. However, the ability of animals to cope with disturbance changes with age and with the accumulation of perturbations over time [43]. To assess the effect of age on energy allocation to growth, future work should analyse temporal dynamics over a longer period, ideally the entire lifespan.

In this study, we used a DLM regression to model the relation between ${\text{CNEA}}_{it - 1}$ and ${\text{CW}}_{it}$ over time. The DLM approach is a powerful tool for analysing time-series variables and enabled allocation coefficient dynamics to be characterized by a stochastic process, without requiring strong deterministic assumptions. With this method, it is possible to determine whether the allocation coefficient increased, decreased, or stagnated, without assuming that it followed any given analytical trend, such as a linear, quadratic, or cubic trend [44]. Our approach takes advantage of the *dlm* package in R [31], which enables processing of the full data with short computation times (around 35 min for the 405,104 measurements). In addition, our simple DLM approach can be expanded to the development of multivariate models or the implementation of fixed (batch, herd, etc.) and random effects [45].

The two-step modelling approach (DLM followed by RR model analysis of the allocation coefficient estimates) allowed interesting results associated with robustness to be identified in a large dataset with daily measurements. Future work should assess the amount and frequency of the data required to apply the developed approach.

### Estimation of the genetic variance of the allocation coefficient $\upalpha _{t}$

We assumed that the “desired allocation” of net energy to growth was driven by two components: the animal's genetic potential and its degree of maturity. In the first step, the objective was to estimate the genetic variance of the allocation coefficient α, as affected by degree of maturity, which changes with age of the pig. To achieve this, we used an RR model to estimate the genetic variance of estimates of ${\upalpha }_{t}$ obtained from the DLM and the slope of the allocation coefficient to growth over time for each individual. Random regression using orthogonal polynomials models have been widely used in genetics, for example to model feed intake or RFI in pigs or rabbits [46, 47]. A random regression of order 1 was chosen to fit the additive genetic and permanent environmental effects, because polynomials of higher order did not significantly improve the model, based on LRT tests. If the end of the measurement period corresponded to a weight closer to mature weight, quadratic random regression may be more suitable [28].

The trait ${\upalpha }_{t}$, which describes the allocation of net energy to growth during the fattening period, had moderate heritabilities, in the same range as those estimated for FCR or RFI. In a previous study [48], we considered the average estimate of ${\upalpha }_{t}$ during the evaluated period and not the daily estimates, and obtained a lower heritability (0.16 ± 0.05) but this was based on a different dataset. For RFI, David et al. [49] reported heritabilities ranging from 0.19 ± 0.06 to 0.28 ± 0.06, using an RR model for weekly estimates of RFI over 10 weeks in pigs.

### Genetic parameters for LSR and production traits

The estimate of the heritability for LSR, which characterizes the environmental variance of ${\upalpha }_{t}$, was low but significantly different from 0. Generally, estimates of the heritability of environmental variance are lower than 0.10 [50] and our estimate for LSR was in the same range as those published for different traits but with a similar REML method, i.e. 012 ± 0.004 for birth weight in rabbits [34], 0.024 ± 0.002 for litter size in pigs [51], 0.029 ± 0.003 to 0.047 ± 0.004 for body weight in broiler chickens [52]. Other studies that were based on the analysis of the log-transformed variance (LnVar) of residuals from the modelling of one time-series variable showed higher heritability estimates than we obtained for LSR, i.e. from 0.20 to 0.24 for milk production [14] and from 0.10 to 0.12 for egg production [15].

Several authors have used the double hierarchical generalized linear model (DHGLM), which allows genetic parameters of the mean of the trait and its residual variance to be estimated in the same structural model [53]. We chose to use a 2-step approach [34, 52, 54] to estimate the genetic parameters for the residual variance rather than a single-step procedure such as DHGLM because it is faster and easier to implement than DHGLM [55], which was beyond the scope of the present study. However, the 2-step approach may underestimate the genetic variance of the residual variance [54], since a homogeneous residual variance between individuals is considered in the first step. In theory, the DHGLM model would make it possible to estimate more accurate EBV [51, 56]. However, Berghof et al. [55] have shown that the two methods provide similar estimates.

Heritability estimates for ADG and RFI were consistent with those reported in the literature for Pietrain or Large-White pigs raised under similar environmental conditions and ranged from 0.33 ± 0.03 to 0.48 ± 0.06 and from 0.21 ± 0.03 to 0.34 ± 0.05, respectively [57, 58]. For carcass traits (BF100 and LD100), the heritability estimates were also consistent with those estimated by Sourdioux et al. [20] and Saintilan et al. [57] in the Pietrain breed (BF100: 0.38 to 0.48; LD100: 0.25 to 0.34). Our estimate of the heritability for FCR was lower than those reported by Saintilan et al. [57], Gilbert et al. [59], and Déru et al. [58], which ranged from 0.30 ± 0.0 to 0.47 ± 0.08.

### Genetic correlations between robustness and production traits

The growth trait ADG was strongly genetically correlated with LSR. Under the current rearing conditions, an animal's ability to be robust, i.e., to have a low LSR value, was strongly genetically linked to its ability to express optimal growth regardless of the environment. Growth has been a major selection trait in the Pietrain breed for over 20 years, and poor growth was a major cause of culling at testing or of non-selection. Nonetheless, even if this genetic correlation estimate was strong, it was significantly different from 1, which implies that, compared to ADG alone, LSR provides additional information on the robustness of these animals. Thus, selection for both traits would result in a greater improvement of the animals’ robustness than selection for growth traits only.

Estimates of the genetic correlations of LSR with the feed efficiency traits FCR and RFI were strong but unfavourable, which could be related to the positive genetic correlation between ADG and FCR, which was affected by the way these two traits were estimated in the present study [18]. Specifically, ADG and FCR were measured over an identical period for all pigs but not standardized for starting and finishing weights. Accordingly, some of the animals tested had reached their mature weight before the test, which led to a drop in FCR and RFI, even though they had previously shown strong growth. Thus, in the present data, there were two types of finisher pigs with low FCR or RFI: those that showed strong growth but did not approach their mature weight during the test period, and those with a low daily feed intake because of low, near maturity, growth [18]. To investigate this, we performed an additional analysis in which we standardized FCR to between 40 and 100 kg, and found that the estimate of its genetic correlation with LSR remained unfavourable but less strong, − 0.34 ± 0.14, while the estimate of its genetic correlation with ADG changed from moderately unfavourable, 0.52 ± 0.06, to close to zero or slightly favourable, − 0.08 ± 0.09. These estimates of the genetic correlation of LSR with FCR and RFI could indicate that the pigs that were the most robust during the test period were not the most efficient ones because they allocated part of their energy to other functions or to maintenance. Indeed, selection for low RFI could impact the ability of the animals to modify their allocation of energy to other functions in order to cope with environmental challenges [59]. This antagonism between short-term efficiency and resilience was put forward by Friggens et al. [16]. In contrast, at the phenotypic level, the correlations of an individual’s average LSR with RFI and FCR were close to 0, − 0.03 and − 0.12, respectively. This suggests that it may not be possible to increase robustness relatively easily without loss of selection response in feed efficiency. In contrast, several studies using divergent selection experiments on RFI showed that animals from the low RFI line (LRFI) adapted better to environmental challenges or at least were not disadvantaged compared to animals from the high RFI line (HRFI). Chatelet et al. [60] showed that the health, growth performance, and feed intake of animals from the LRFI line were less impacted by poor hygienic conditions than those of animals from the HRFI line. In the same selection experiment, the risk of being culled between 70 days of age and slaughter was 1.8 times lower in the LRFI line than in the HRFI line [59]. In another selection experiment, Dunkelberger et al. [61] suggested that pigs from the LRFI line were more robust to porcine reproductive and respiratory syndrome virus (PRRSV) challenges, as their growth and health were less affected. These results seem to contradict the resource allocation theory and the genetic correlations estimated in our study. However, this study was carried out on the Pie NN line, which is a sire line, while the selection experiments on RFI were done on animals from the Large-White (or Yorkshire) breed, which is a dam line. The Pietrain sire line has been selected for several generations to improve feed efficiency, growth, and carcass characteristics, potentially to the detriment of other traits, such as robustness. Due to the different breeding objectives, there may be a different allocation pattern of resources in this line compared to the maternal lines used in the RFI selection experiments.

Estimates of genetic correlations between robustness and BF100 were slightly unfavourable. We hypothesize that the capacity to be robust could be associated with having greater body reserves, allowing the animal to face perturbations. This result is not consistent with those of van Milgen and Noblet [42] who reported that the mobilization of body reserves occurs rarely in growing animals. One hypothesis is that the latter may be true under non-limiting environmental conditions but that mobilization during the growth phase is deployed to deal with environmental perturbations.

### Relationships of LSR EBV classes with other phenotypes

Our study shows that modelling the longitudinal energy allocation coefficient to growth offers the opportunity to develop a proxy for robustness that is heritable. However, this proxy has to provide benefits to pig farmers, i.e., it should be able to identify animals that cope well with environmental disturbances. In practical terms, these are animals that receive fewer treatments and are presented for testing in good health. Analysis of relationships of LRS EBV quartiles with phenotypes that are routinely collected on farms showed the most favourable relationships for the most robust animals, i.e., those from the quartile with the lowest EBV for LSR (Q1). Thus, in spite of its low heritability, including LSR in the breeding goal would be an opportunity to improve the robustness qualities of the Pie NN line during the fattening period. However, in the present study, LSR was evaluated over only a short period of the animal’s life and it would be desirable to investigate the effects of selection on EBV for LSR based on the whole lifespan of relatives (dam, sire, purebred or crossbred offspring). In addition, the relationship of LSR with reproductive performance of boars (sperm production) and females (fertility, productive longevity, survival) should be investigated.

### Environmental conditions

This study was carried out in a higher biosecurity environment than that of regular farms, which is because a breeding company needs to minimize risks for a purebred nucleus. The other environmental conditions (feed characteristics, barn design, density, etc.) were close to those found on production farms in France that are designed to minimize exposure to environmental challenges. When designing a selection program, it is necessary to maintain a balance between conditions that allow full expression of performance while meeting sanitary requirements versus conditions that favour expression of robustness. Although purebred nucleus environments are qualified as favourable, the animals are subjected to stresses that can be chronic, such as social stress or heat waves. Rearing animals under challenging conditions allows for better phenotyping of the robustness [62]. The antagonism of conditions to evaluate robustness versus production potential may be partly overcome by the use of short-term challenges, such as feeding challenges. Indeed, offspring of these purebred pigs will likely be reared in harsher and more variable environments that favour expression of robustness. This relationship between robustness and diverse rearing conditions can, however, not be dissociated from genotype × environment (G×E) interactions [63], which may cause reranking of sires and has a greater impact on traits based on variances than on traits based on means [15]. The acquisition of data on relatives of selection candidates that are reared on farms equipped with AFS allows evaluation of the effects of G×E interactions.

In this study, we proposed an approach to characterize robustness based on variability in allocation coefficients to growth of fattening pigs. However, when studying the allocation pattern, it is important to also assess the acquisition trajectory [16, 64] because an increase in energy for a function such as health can be achieved not only by changing allocation but also by increasing the overall acquisition of energy. In a routine selection approach, it would be relevant to add to LSR a trait that characterizes variability in energy acquisition.

## Conclusions

The trait LSR can be interpreted as an indicator of the response of the animal to perturbations/stress, i.e. as a proxy for robustness. This study shows that LSR has a low heritability but may be amenable to selection. We found that LSR is favourably genetically correlated with growth rate and unfavourably genetically correlated with feed efficiency (FCR and RFI). The favourable association of LSR EBV with treatment with antibiotics or anti-inflammatories and with health issues showed that selection on LSR could have a positive impact on the use of antibiotics and on animal welfare.

## Data Availability

The datasets analysed in this study are not publicly available because they are part of the commercial breeding program of AXIOM. However, they are available from the corresponding author on reasonable request.

## Appendix

### Appendix: List of individual observations performed during the individual test from Lenoir et al. [18]

**Table:**

	Observation
Observations taken into account to define the robustness traits	Abscess
	Cannibalism
	Hygroma (Capelet)
	Weak development/low body condition
	Callus
	Shortness of breath
	Necrotic ear
	Out of test (testing body weight < 70 kg)
	Shaker
Observations not taken into account to define the robustness traits	Lack of leg soundness
	Low and short
	Conformation/body development
	Double-muscled (Culard)
	Important conformation
	Fat animal
	Asymmetric hooves
	Teats default
	Incorrect conformation
	Hernia
